# COVID-19 and Alzheimer’s disease: Impact of lockdown and other restrictive measures during the COVID-19 pandemic

**DOI:** 10.17305/bb.2023.9680

**Published:** 2024-04-01

**Authors:** Ahmed Daniyal Nawaz, Mohammad Zulqurnain Haider, Saghir Akhtar

**Affiliations:** 1 College of Medicine, QU Health, Qatar University, Doha, Qatar

**Keywords:** Coronavirus disease 2019 (COVID-19), severe acute respiratory syndrome coronavirus 2 (SARS-CoV-2), Alzheimer’s disease, lockdown, restrictions, isolation measures, dementia, neurological symptoms, caregivers

## Abstract

Severe acute respiratory syndrome coronavirus 2 (SARS-CoV-2) infection initially results in respiratory distress symptoms but can also lead to central nervous system (CNS) and neurological manifestations, significantly impacting coronavirus disease 2019 (COVID-19) patients with neurodegenerative diseases. Additionally, strict lockdown measures introduced to curtail the spread of COVID-19 have raised concerns over the wellbeing of patients with dementia and/or Alzheimer’s disease. The aim of this review was to discuss the overlapping molecular pathologies and the potential bidirectional relationship between COVID-19 and Alzheimer’s dementia, as well as the impact of lockdown/restriction measures on the neuropsychiatric symptoms (NPS) of patients with Alzheimer’s dementia. Furthermore, we aimed to assess the impact of lockdown measures on the NPS of caregivers, exploring its potential effects on the quality and extent of care they provide to dementia patients. We utilized the PubMed and Google Scholar databases to search for articles on COVID-19, dementia, Alzheimer’s disease, lockdown, and caregivers. Our review highlights that patients with Alzheimer’s disease face an increased risk of COVID-19 infection and complications. Additionally, these patients are likely to experience greater cognitive decline. It appears that these issues are primarily caused by the SARS-CoV-2 infection and appear to be further exacerbated by restrictive/lockdown measures. Moreover, lockdown measures introduced during the pandemic have negatively impacted both the NPSs of caregivers and their perception of the wellbeing of their Alzheimer’s patients. Thus, additional safeguard measures, along with pharmacological and non-pharmacological approaches, are needed to protect the wellbeing of dementia patients and their caregivers in light of this and possible future pandemics.

## Introduction

Coronavirus disease 2019 (COVID-19) was declared a global pandemic on February 11, 2020, and was caused by a novel strain of coronavirus later identified as severe acute respiratory syndrome coronavirus 2 (SARS-CoV-2) [1]. As of October 31, 2023, over 771 million confirmed cases of COVID-19, including nearly 7 million deaths, have been reported to the World Health Organization (WHO) [2]. Similar to the previously identified SARS-CoV-1 virus, SARS-CoV-2 utilizes the angiotensin-converting enzyme 2 (ACE-2) receptor for cellular entry [3, 4]. ACE-2 is highly expressed in the lungs, which is why COVID-19 predominantly presents as a respiratory-related illness. However, it can rapidly affect other organs that express ACE-2 receptors, leading to multi-organ disease [5]. ACE-2 receptors are also present in brain tissues, including neurons, potentially enabling the virus to enter the central nervous system (CNS). This could lead to the central neurological manifestations observed in COVID-19.

As a result of the pandemic, extreme measures were undertaken to curb the spread of COVID-19, such as social distancing, lockdown restrictions, limiting the number of visitors in restaurants, hospitals, and care homes, closing schools and non-essential businesses, reducing the number of people working on site and implementing nighttime curfews. These anti-social lockdown measures likely led to worsening neurological symptomology, including cognitive impairment, particularly in older adults, especially those suffering from dementia [6–8]. For example, Wang et al. [9] observed a worsening of neuropsychiatric symptoms (NPS), such as anxiety, depression, irritability, and apathy in patients with Alzheimer’s dementia during the first five weeks of lockdown. This finding highlights the significant negative impact that lockdown measures have on individuals with Alzheimer’s dementia.

Furthermore, lockdown measures were also reported to negatively impact the NPSs of caregivers of dementia patients. This impact compromised their ability to accurately assess the wellbeing of these patients and could potentially affect the degree and quality of care provided by the caregivers to their patients [10, 11]. Therefore, it is crucial to further explore the links between COVID-19 and NPS in both dementia patients and their caregivers. A better understanding of clinical symptomatology is essential for the provision of appropriate disease management strategies. The most common type of dementia is Alzheimer’s dementia [12]. This is a neurodegenerative disorder mainly affecting the hippocampus which is directly responsible for memory, cognition, and learning [11]. Furthermore, recent studies have shown that over a quarter of COVID-19 patients developed neurological symptoms, such as memory loss and confusion, possibly arising from a cytokine storm caused by the SARS-CoV-2 infection leading to neuroinflammation, a pathological feature shared by Alzheimer’s dementia [13, 14].

In this review, we aim to discuss the common molecular pathological features and the potential bidirectional clinical relationship between COVID-19 and dementia. Additionally, we will explore the impact of lockdown/restriction measures on the NPS of dementia patients. Furthermore, we intend to assess the impact of lockdown measures on the NPS of caregivers and discuss how this might influence the degree and quality of care provided to dementia patients.

## Review methodology

The PubMed database was searched for articles using the terms: COVID-19, dementia, Alzheimer’s disease, lockdown, and caregivers. The Google Scholar database was also screened for relevant articles. All potential articles were then exported to Rayyan QCRI software for analysis to determine inclusion or exclusion. The inclusion criteria for the research were: (1) studies on COVID-19 and neurological symptoms, (2) articles linking COVID-19 infection with Alzheimer’s patients, and (3) full-text articles available in English. The exclusion criteria included case report studies and studies not involving dementia/Alzheimer’s disease patients.

## Common pathological features between COVID-19 and Alzheimer’s disease

The impact of COVID-19 on the lungs is caused by a complex molecular pathogenesis that involves interactions between the virus and the human respiratory system. The majority of COVID-19 patients experience mild flu-like symptoms, such as fever, cough, rhinorrhea, general malaise, and loss of taste and smell which are brought on by the virus [15]. However, a small percentage of individuals develop severe respiratory distress arising from the ensuing acute lung injury (ALI), such as pneumonia, which is typically accompanied by coagulopathy and can require mechanical ventilation in extreme cases. The majority of the patients with severe symptomology are elderly males [16]. SARS-CoV-2’s entry into the lung starts when the viral spike protein, which is prominently present on the virus’s surface, recognizes and interacts with ACE2 receptors present on the host cell membrane highlighting the crucial role played by the ACE2 receptor in viral entry via the pulmonary system [17, 18]. What follows is a series of complex molecular steps that ultimately results in the production of further viral particles which are released to infect nearby healthy cells to sustain the infection within the respiratory system and beyond. Current data suggests that the failure of the immune system to regulate and restrict SARS-CoV-2 lung infection may be the cause of these clinical characteristics [19].

The delicate balance of the alveolar-capillary barrier, which separates the alveoli from the blood vessels, can be disrupted by the inflammatory response [20]. The buildup of immune cells and fluid in the alveolar gaps can result in the development of pulmonary edema, which affects the ability of the lungs to exchange oxygen and carbon dioxide. Acute respiratory distress syndrome (ARDS), which is characterized by extensive lung damage and hypoxia, can occur in severe cases of COVID-19. The ensuing inflammation and lung tissue damage may eventually impair respiratory function and cause a life-threatening respiratory failure [21].

Most of the studies on COVID-19 have aimed to discover the pathological processes that take place in the lungs and link them to the severity of the disease. However, the neurological symptoms that people suffer from have mostly been ignored. Neurological manifestations of COVID-19 range from headache, loss of smell, confusion, strokes, brain hemorrhage to memory loss [22]. Increasing evidence shows that SARS-CoV-2 can infect neurons and affect brain function through chronic hypoxia, metabolic dysfunction, systemic inflammation, and immune dysregulation [23–25]. A retrospective study performed in China on 214 COVID-19 patients showed that almost 60% of patients suffered from neurological symptoms, such as dizziness, headache, ataxia, vision, taste, and smell impairment [6]. If neurological symptoms occur in such a high number of COVID-19 patients, it might be expected that neurological symptoms in patients with both COVID-19 and dementia would be even higher [26].

**Figure 1.**
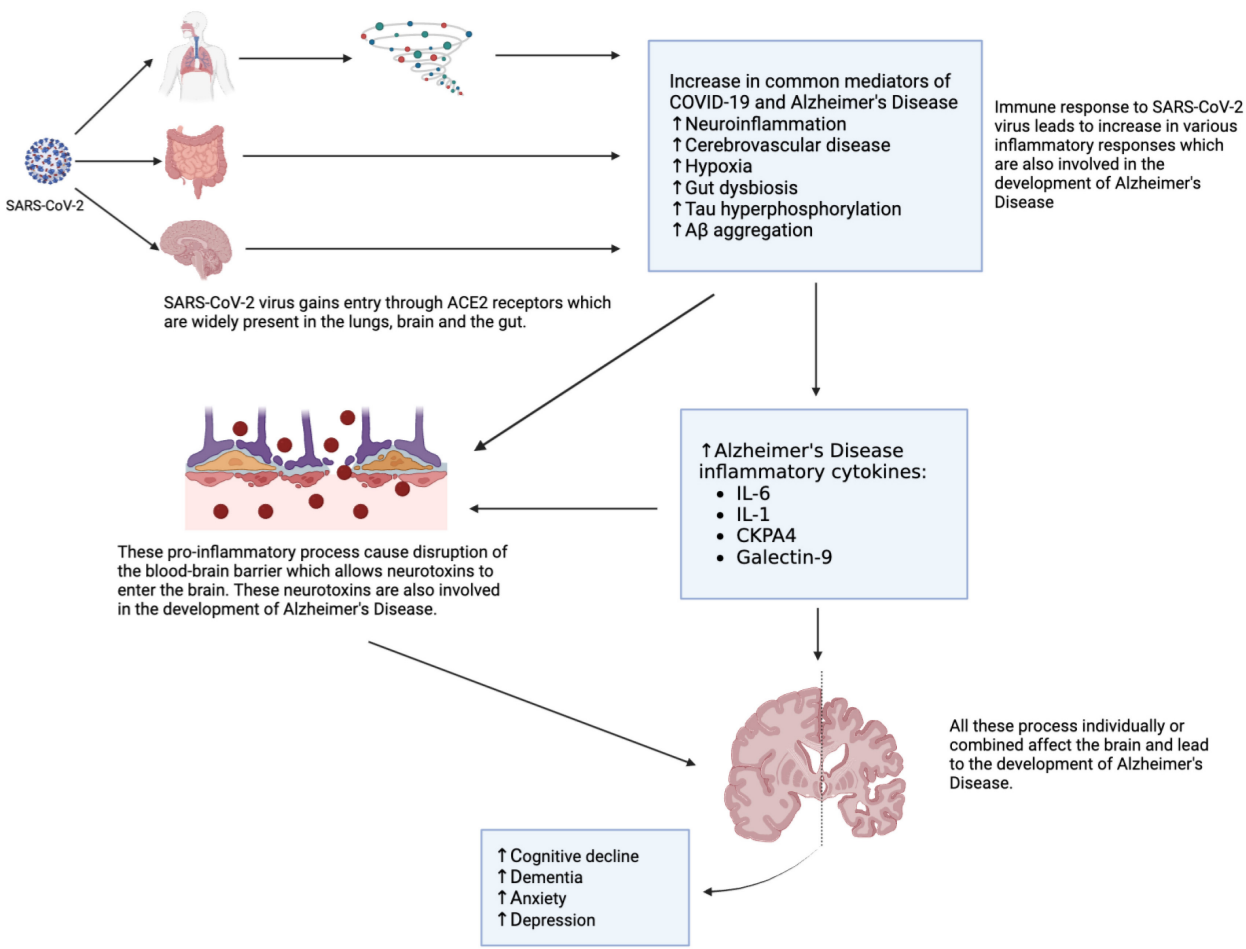
**Key molecular links between COVID-19 and Alzheimer’s disease pathology.** The SARS-CoV-2 virus enters the human body through ACE2 receptors expressed in the lung, gut, brain, and potentially other organs [17, 54]. COVID-19 can also increase expression of ACE2 receptors which may further increase entry of more SARS-CoV-2 [101]. The entry of the virus in the lungs can result in a cytokine storm that gives rise to several pro-inflammatory processes [34]. The resulting cytokine storm and inflammation can lead to cerebrovascular dysfunction that can consequently cause hypoxia. Chronic hypoxia can accelerate hyperphosphorylation of tau protein, a hallmark of pathogenesis in Alzheimer’s dementia [47]. The aggregation of beta-amyloid (Aβ) triggered by the SARS-CoV-2 could also potentially be a mechanism through which COVID-19 elevates the risk of acquiring Alzheimer’s disease, leading to the accumulation of Aβ in neurons and the subsequent loss of synapses [37]. COVID-19 can also lead to neuroinflammation which results in increased tau hyperphosphorylation [41]. These pro-inflammatory processes give rise to several cytokines, such as IL-1, IL-6, CKPA4, and Galectin-9 which are involved in the development of Alzheimer’s disease [35]. These pro-inflammatory processes and cytokines can lead to the development of Alzheimer’s disease. However, they also lead to endothelial dysfunction of blood–brain barrier, allowing neurotoxins to enter the brain and further increasing the risk of Alzheimer’s disease [49]. COVID-19 infection also causes gut dysbiosis which can also potentially cause Alzheimer’s disease [51]. Thus, there is much overlap in the pathology of COVID-19 and Alzheimer's diease (see main text for further details). SARS-CoV-2: Severe acute respiratory syndrome coronavirus-2; ACE-2: Angiotensin-converting enzyme 2; COVID-19: Coronavirus disease 2019; IL: Interleukin; CKAP4: Cytoskeleton-associated protein 4. Figure 1 was made with BioRender.com.

There is also robust evidence showing common cross-talk between Alzheimer’s disease and COVID-19’s in terms of their neurological symptoms. [27]. ACE2 receptors are widely distributed in the neurons, ganglia, and brain vascular endothelium and are highly elevated in Alzheimer’s disease patients, especially in the hippocampus and cerebral cortex [28]. In addition, SARS-CoV-2 can bind to ACE2 receptors in the olfactory bulb and give rise to severe innate immune responses which can lead to neuroinflammation and endothelial dysfunction of the blood–brain barrier [29, 30]. This dysfunction may allow circulating neurotoxins to enter the brain and cause neurological damage and consequently lead to neuronal death, especially in the hippocampus and cerebral cortex [31, 32]. The idea that Alzheimer’s dementia, even before the arrival of SARS-CoV-2, may be caused by an infectious agent had been proposed for decades and has received some support but largely remains controversial [33]. In addition to the increased risk of COVID-19 infection in Alzheimer’s dementia patients, it has been demonstrated recently that SARS-CoV-2 infection produces long-lasting neurological sequalae, which may be associated with inflammatory processes central to Alzheimer’s dementias neuropathology [34] (Figure 1).

A major link between Alzheimer’s disease and COVID-19 is the common inflammatory mediators that appear to be elevated in both conditions. It has been observed that interleukin (IL)-1, IL-6, cytoskeleton-associated protein 4 (CKAP4), and galectin-9 are all increased in both Alzheimer’s disease and COVID-19 [35]. These cytokines operate as chemical signals by attracting immune cells, such as macrophages and T lymphocytes to the infection site leading to the formation of inflammatory infiltrates [36]. There are also concerns that patients who have recovered from COVID-19 may be at an increased risk of developing Alzheimer’s disease. In a retrospective cohort study involving over 6 million adults aged 65 years and older, those with a history of COVID-19 were found to be at a significantly increased risk of receiving a new diagnosis of Alzheimer’s disease within 360 days post-diagnosis (hazard ratio [HR] 1.69, 95% confidence interval [CI] 1.53–1.72), with the risk being particularly elevated in individuals aged 85 years and older, and in women [34]. It is interesting to note that while elderly males are more susceptible to acquiring either Alzheimer’s or COVID-19 alone, post-COVID-19 diagnosis, the development of newly diagnosed Alzheimer’s dementia appears to be more prevalent in elderly females. The reasons for this gender-specific trend are still unclear and warrant further investigation.

Since the development of Alzheimer’s disease is a multifactorial and complex process, its etiology is mostly unknown. In addition to the idea that Alzheimer’s disease may be caused by an infectious agent, there are several other theories explaining the underlying molecular mechanisms of Alzheimer’s disease, as well as its possible overlapping connection with COVID-19 pathology. The interlinking molecular dynamics between COVID-19 and Alzheimer’s disease may have clinical implications and could offer potential novel interventional strategies for the treatment of COVID-19 patients at risk for developing Alzheimer’s disease.

## The amyloid hypothesis in Alzheimer's disease and its potential cross-talkk with COVID-19

The amyloid hypothesis in Alzheimer’s disease pathology asserts that accumulation of beta-amyloid plaques results in neuroinflammation and neuronal dysfunction, especially in the hippocampus which is often times the first and most affected region of the brain in Alzheimer’s disease [37]. It is also thought that the inflammatory cascade brought on by COVID-19 may enhance beta-amyloid aggregation and neuroinflammation, perhaps hastening the onset of Alzheimer’s disease because of beta-amyloid pathway dyshomeostasis [37]. Another potential way COVID-19 could impact Alzheimer’s disease is through the degradation of the SARS-CoV-2 spike protein by endogenous proteases that might lead to the subsequent creation of amyloid fibrils [38, 39].

Since the SARS-CoV-2 spike protein is the primary target of COVID-19 vaccinations, antibodies that bind to it may be able to prevent the cellular entry of SARS-CoV-2 and the subsequent degradation of its spike protein that would otherwise lead to the production of amyloid fibrils [31]. Thus, immunization against COVID-19 might represent a particularly attractive preventative option to mitigate the possible injurious infection effects on the brain, including cognitive decline [33] and the potential onset of Alzheimer’s dementia. This notion is further supported by the findings that past exposure to vaccines against diphtheria or tetanus, poliomyelitis, and influenza likely protects against subsequent development of Alzheimer’s dementia [33], though the exact underlying mechanisms of protection are not fully understood.

## Tau hypothesis in Alzheimer's disease and its potential cross-talkk with COVID-19

The tau hypothesis emphasizes the aberrant build-up of hyperphosphorylated tau protein and the formation of neurofibrillary tangles that represent another hallmark of Alzheimer’s dementia [40]. Recent studies have suggested that tau hyperphosphorylation may be influenced by neuroinflammation, which can be brought on by any viral infection, presumably COVID-19 [41]. It is possible that COVID-19-related inflammatory processes and oxidative stress may enhance tau pathology, connecting the viral infection to the mechanisms underlying Alzheimer’s disease [42]. It was also shown that SARS-COV-2 infection led to oxidative stress overload and enhanced TGF-β signaling ultimately resulting in hyperphosphorylation of tau, thereby providing a direct link between COVID-19 and Alzheimer’s disease [43]. Thus, COVID-19 neuropathology overlaps with some Alzheimer’s disease-like features that could potentially serve as therapeutic targets for amelioration of cognitive defects associated with both diseases.

## Cerebral vascular dysfunction theory in Alzheimer’s disease and linkk to COVID-19

Cerebrovascular dysregulation is one of the earliest detectable changes in Alzheimer’s disease etiology that precedes the descent into dementia [44]. Ischemic events, such as stroke and silent infarcts trigger cerebrovascular dysfunction, accelerate cognitive decline, and increase dementia risk [45]. Furthermore, the resulting decrease in cerebral blood flow can induce the formation of amyloid β [44].

Systemic infections are known to alter brain cytokine levels, exacerbate cerebral hypoperfusion, and cause blood–brain barrier leakiness associated with Alzheimer’s dementia [46]. Thus, systemic infection is an important contributor to dementia, requiring early identification and treatment in the elderly population [46]. As discussed earlier, one of the hallmarks of COVID-19 is a cytokine storm and systemic inflammation, which can lead to cerebrovascular dysfunction and hypoxia [47]. Hypoxia has been shown to accelerate hyperphosphorylation of tau protein, a key hallmark in the pathogenesis of Alzheimer’s dementia [48]. Moreover, hypoxia and vascular dysfunction lead to a disrupted and dysregulated blood–brain barrier further exacerbating neuroinflammation which has been directly linked to Alzheimer’s dementia as well as indirectly through increased production of beta-amyloid plaques [49]. Thus, there appears to be an important overlap in the disruption of cerebral vascular flow between COVID-19 and Alzheimer’s dementia that may represent a common target for potential therapeutic intervention.

## Gut flora dysbiosis theory in Alzheimer’s disease and COVID-19

The gut microbiome is known to be an important and key regulator of inflammation in the body including the brain [50]. A growing body of research shows that the composition of the gut microbiota is altered in Alzheimer’s disease and points to dysbiosis of the gut microflora as being another key hallmark in the development of Alzheimer’s dementia [51]. Verhaar et al. [52] reported Alzheimer’s dementia is associated with a reduced population of *Faecalibacterium, Eubacterium*, *Anaerostipes*, and *Ruminococcus* as compared to the control group. Moreover, an increase in *Phascolarctobacterium* and a decrease in *Bacteroides* was reported in the prodromal stages of Alzheimer’s disease [51]. These gut modifications may have an impact on the synthesis of bacterially produced metabolites such as short-chain fatty acids, which have been proven to have neuroprotective properties [53].

Although COVID-19 is largely understood to be a respiratory infection, its effects on the gut flora and gastrointestinal symptoms are also becoming more established. It has been demonstrated that the virus enters the digestive system via ACE2 receptors, which are highly expressed in the gut epithelium [54]. The presence of SARS-COV-2 in the GI tract has the potential to alter the composition and operation of the gut microbiota, leading to COVID-19-induced gut dysbiosis. The gut–brain axis is one potential link between gut dysbiosis brought on by COVID-19 and observed in Alzheimer’s disease. Similar to the findings in Alzheimer’s patients, several studies reported COVID-19 patients exhibit gut dysbiosis with a decrease in Bacteroides, *Faecalibacterium, and Ruminococcus*i along with an increase in *Phascolarctobacterium* [50, 55, 56]. Since SARS-COV-2 viral-induced dysbiosis leads to alterations in the gut microflora that mirror some of the microbiota changes observed in Alzheimer’s disease, it might offer a rational explanation for why COVID-19 patients are predisposed to Alzheimer’s disease.

## Clinical outcomes of COVID-19 infection in patients with dementia

An important clinical correlation to investigate is the bidirectional link between COVID-19 and dementia in terms of clinical outcomes, such as morbidity and mortality. Key questions are whether having dementia increases your probability of acquiring COVID-19 and whether COVID-19 leads to worsening morbidity and mortality within dementia patients. A study was conducted with a population of 61 million adults which included 1 million dementia patients, 15,770 COVID-19 patients, and 810 patients with both dementia and COVID-19 [9]. The authors investigated the following outcomes: COVID-19 diagnosis, death, and 6-month hospitalization percentages among this population. Patients with dementia were found to have 2-fold greater odds of acquiring COVID-19 as compared to non-dementia patients (odds ratio [OR] 2.00, 95% CI 1.94–2.06, *P* < 0.001) highlighting the role that dementia plays as an independent risk factor in contracting COVID-19 [9]. The underlying reasons for this correlation are not fully understood. However, a possible explanation might be the inability of patients with Alzheimer’s disease to comply with safeguarding protocols due to their intrinsic cognitive impairment. The propensity to develop COVID-19 due to their cognitive impairment might be exacerbated by the close proximity in which these patients were living in care homes and represents an additional risk factor for acquiring SARS-COV-2 infection [57].

Moreover, in a large-scale study involving more than 1 million patients, the 6-month mortality rate of COVID-19-infected dementia patients was found to be 16% higher than that of only COVID-19 patients (*P* < 0.001) and 13% (*P* < 0.001) [9]. Other multiple studies have also reported that COVID-19 dementia patients had two to five times greater odds of mortality as compared to patients who only had either COVID-19 or dementia, highlighting that COVID-19 and dementia both play as independent risk factors in mortality [58, 59].

The effects of having COVID-19 in dementia patients as compared to non-dementia COVID-19 patients are not just limited to worsening mortality. Studies have also shown worsening clinical outcomes such as a 30%–50% increased risk of hospitalization and ICU admission in COVID-19 dementia patients [9, 60]. COVID-19 dementia patients also exhibited five times greater odds of facing early, late, and progressive cognitive decline as compared to COVID-19 patients without dementia [61, 62].

In contrast, Vekaria et al. [63] reported no significant difference in mortality rates, 90-day admission rates and length of hospital stay in COVID-19 dementia patients as compared to COVID-19 patients without dementia, though, they did report dementia to be the main predictor for ventilator use length in COVID-19 patients. In general, with few exceptions, studies generally imply an increasing probability of acquiring COVID-19 in dementia patients along with facing worsening morbidity and mortality in patients with both COVID-19 and dementia as compared to patients with only one of the two conditions (Table 1).

**Table 1:** A summary of the key clinical studies showing the bidirectional impact of COVID-19 and Alzheimer’s disease and the impact of isolation/restrictive measures on the wellbeing of dementia patients and their caregivers

**Author**	**Study design**	**Study population**	**Measure of outcome**	**Outcome**	**Reference**
**SARS-CoV-2 and Alzheimer’s disease**		
Wang et al. (2021)	Retrospective case-control	61,900,000	Risks, disparity, and outcomes for COVID-19 in patients with dementia	Patients with dementia were at increased risk for COVID-19 compared to patients without dementia (adjusted odds ratio 2.00).	[9]
Kim et al. (2021)	Cohort	5349	Evaluating the increased mortality risk within 14 days of COVID-19 diagnosis in dementia patients	The mortality rate within 14 days after COVID-19 diagnosis in dementia patients and the controls was 23.7% vs 1.7%, respectively.	[59]
Vekaria et al. (2022)	Descriptive study	10,473	The role of patients’ baseline characteristics specifically, dementia, in determining overall health outcomes in COVID-19 patients	Higher mortality was observed in dementia group 30.8% vs 26.4% in non-dementia group.	[63]
Bianchetti et al. (2020)	Retrospective study	627	Mortality in COVID-19 patients with dementia and COVID-19 patients without dementia	Adjusted odds ratio for fatality in COVID-19 patients with dementia and COVID-19 patients without dementia was 1.84 (95% CI 1.09–3.13, *P* < 0.05).	[58]
Wang et al. (2022)	Retrospective cohort	6,245,282	Risk of development of Alzheimer's disease post-COVID-19 infection in adults (age > 65)	COVID-19 patients had 1.69 times (95% CI 1.53–1.72) higher risk of developing Alzheimer’s disease compared to patients without COVID-19.	[34]
**Isolation and dementia**					
Boutoleau-Bretonnière et al. (2020)	Descriptive study	38	Effects of confinement during COVID-19 on neuropsychiatric symptoms in patients with Alzheimer's disease	26.3% of patients demonstrated neuropsychiatric changes during confinement. Mini-Mental State Examination (MMSE) was worse for these patients compared to those who did not have neuropsychiatric changes.	[100]
El Haj et al. (2020)	Descriptive study	58	The effects of measures against COVID-19 on the mental health of participants with Alzheimer’s disease in retirement homes	Participants reported higher depression (*P* ═ 0.005) and anxiety (*P* ═ 0.004) during COVID-19 crisis than before the crisis.	[64]
El Haj et al. (2016)	Descriptive study	46	Relationship between social isolation, loneliness, and hallucinations in patients with Alzheimer’s disease	Patients with Alzheimer's disease showed higher levels of hallucinations, loneliness, and social isolation (*P* < 0.001) than healthy controls.	[65]
Lara et al. (2020)	Descriptive study	40	Effect of lockdown on the neuropsychiatric symptoms of patients with Alzheimer’s disease	The mean (SD) total baseline neuropsychiatric inventory (NPI) score was 33.75 (22.28), compared with 39.05 (27.96) after confinement (*P* ═ 0.028). 30% of patients and 40% of caregivers reported a worsening of the patients’ health status during confinement.	[85]
Gan et al. (2021)	Retrospective descriptive study	205	Investigating the cognitive and neuropsychologic changes in cognitive impairment patients, as well as the proportions of rapid cognitive decline (RCD) before and during the COVID-19 pandemic	Alzheimer's disease patients during the COVID-19 pandemic were 0.408 times (95% CI 0.232–0.716) less likely to suffer rapid cognitive decline than the control.	[66]
**Care homes and SARS-CoV 2 infection**					
Chen et al. (2021)	Descriptive study	177	The impact of lockdown on cognitive function and neuropsychiatric symptoms over a 1-year follow-up period in patients	42% of mild cognitive impairment, 54.3% of Alzheimer's disease, and 72.7% of dementia with Lewy bodies patients had a decline in MMSE scores and 54.4% of dementia with Lewy bodies patients had worsening NPI scores.	[67]
Hwang et al. (2021)	Descriptive study	32	Assessing the influence of COVID-19 on concerns of current family caregivers of patients with dementia	Over 70% of the study participants reported worrying about spreading COVID-19, 41% reported they had taken on additional caregiving duties for others in their family since COVID-19, and 62% reported one or more anxiety symptoms.	[11]

COVID-19: Coronavirus disease 2019; SARS-CoV-2: Severe acute respiratory syndrome coronavirus 2.

## The impact of lockkdown and other restrictive measures on patients with dementia

An important question is whether the lockdown measures introduced during COVID-19 pandemic had an impact on the NPSs of Alzheimer’s disease patients. It is reported that almost 25% of patients with Alzheimer’s disease had worsening NPS changes during lockdown, with the duration of confinement acting as an independent risk factor for the severity of NPS changes [64]. Moreover, several studies reported the changes in mood symptoms of dementia patients as a result of the confinement during COVID-19 lockdown measures [61–65]. For example, a study assessed 58 patients with Alzheimer’s disease and found that all patients had worsening depression (*P* < 0.05) and anxiety (*P* < 0.04) during the pandemic as compared to pre-pandemic levels. Others have reported worsening depression, anxiety, hallucinations, loneliness agitation, and aberrant motor behaviors in patients with Alzheimer’s dementia during lockdown [65–69].

The adverse effect of the lockdown was also seen on the cognitive abilities of patients with dementia. A meta-analysis by Suarez-Gonzales et al. which included 15 studies and almost 6400 patients reported that 60% of the studies found a significant negative change in cognition along with 93% of the studies reporting worsening or new onset of behavioral and psychological symptoms, such as apathy, anxiety, and depression [70]. Similar results were observed in a subsequent study of 113 dementia patients, who were reported to have experienced a greater rate of decline in their memory and recall abilities [71].

These studies point out that lockdown measures during the pandemic led to a steeper decline in the NPS of patients suffering from Alzheimer’s dementia. Patients with Alzheimer’s disease suffer a worsening of motor symptoms, such as depression, anxiety, apathy, agitation, and hallucinations along with facing cognitive decline in the domain of memory, recall, and communication. Interestingly, these findings were observed in dementia patients irrespective of whether they were living in their own homes or nursing homes [68].

However, a minority of studies have presented findings that diverge from those discussed above. A few studies have shown that cognitive differences between dementia patients in lockdown as compared to a control group in pre-lockdown had no significant difference. In a study of 2015 cognitively impaired patients, Gan et al. reported that patients with Alzheimer’s disease during the COVID-19 pandemic were 0.408 times (95% CI 0.232–0.716) less likely to suffer rapid cognitive decline (RCD) than the control [66]. Moreover, another study of 60 patients with Alzheimer’s disease reported no changes in the functional status of the patients (*P* ═ 0.14) according to the clinical dementia rating (CDR) score during the pandemic [69]. In general, with some exceptions, the majority of the studies point to a similar conclusion, that the lockdown measures introduced during the COVID-19 pandemic led to a steeper decline in the NPS of patients suffering from Alzheimer’s dementia (Table 1).

## The impact of lockkdown and restrictive measures on caregivers of patients with dementia

It is now clear that lockdown and restriction measures introduced during the pandemic have had detrimental effects on NPS of patients with Alzheimer’s disease. The question arises as to what is the impact of such measures on the caregivers of these patients. Caregivers were assessed to have had a significantly increased burden of work along with worsening psychological states during the lockdown [72]. One study found that around 40% of Alzheimer’s disease caregivers experienced an increased caregiver burden, whereas around 30% experienced worsening anxiety and depression during the lockdown [67]. Similarly, another study reported that 60% of the 34 caregivers studied experienced worsening anxiety symptoms during the pandemic. Furthermore, those caregivers who reported anxiety symptoms also reported lower functional independence scores for their Alzheimer’s disease patients, compared to caregivers who did not report anxiety symptoms (*P* < 0.036) [11]. This highlights that not only did the lockdown worsen the NPS of caregivers but it also negatively impacted their ability to assess the wellbeing of their patients and then as a result to provide adequate and proper treatment. Similar findings were reported by Quinn et al. [73], where 50% of 242 caregivers reported loneliness and almost 45% reported being “trapped” due to the non-availability of alternative caregivers to cover their duties. This highlighted the excessive burden on caregivers and their perception of experiencing increased stress at work during the pandemic [74, 75]. However, despite the obvious greater stress of caregivers at work during the lockdown, having social support, close relationships with family and friends, interests, and hobbies were deemed beneficial in mitigating the emotional and psychological challenges brought on by the SARS-CoV-2 pandemic and lockdown [76]. This was further supported by findings showing that caregivers experienced increased optimism and minimal impact on their duties, attributed to coping mechanisms they utilized. These included positive thinking, emotional and informational support, as well as venting coping mechanisms, all of which helped to reduce their stress levels [72].

## Perspectives

Several theories have been postulated to show a mechanistic link between COVID-19 and Alzheimer’s dementia. SARS-COV-2 virus, whilst predominantly affecting the respiratory system, also affects the CNS system through the presence of ACE-2 receptors in the brain [9, 21, 77]. Neuroinflammation appears to be the underpinning pathophysiology of COVID-19, which can directly and/or indirectly crosstalk with the pathogenesis of Alzheimer’s disease, through several potential mechanisms, including beta-amyloid plaque formation, tau hyperphosphorylation, and cerebral vascular dysfunction [37, 41, 49, 78].

Dysbiosis of the commensal intestinal microbiota and their metabolites appears to be another common pathological feature of COVID-19 and Alzheimer’s disease. However, the precise mechanisms of chemical and molecular crosstalk are poorly understood [79]. For example, it has been shown that elevated levels of trimethylamine N-oxide (TMAO) are associated with the pathogenesis of Alzheimer’s disease and are known to result in increased formation of reactive oxygen species and pro-inflammatoy cytokines [80, 81]. Production of TMAO is known to be associated with the increased population of *Ruminococcus* bacteria in Alzheimer’s patients and also been found to be elevated in the gut flora of COVID-19 patients [82, 83] providing a potential common link between the two conditions. However, the precise role of *Ruminococcus* bacteria and TMAO along with other microbiome metabolites need to be studied in more depth to decipher a possible interplay and mechanistic links between SARS-CoV-2 and Alzheimer’s disease. Furthermore, the additional impact of gut dysbiosis resulting from co-morbidities, such as hypertension and diabetes in SARS-CoV-2 infection remains to be elucidated [79]. Thus, further research is necessary to understand the precise impact of each pathology on the bidirectional relationship. Identifying any convergent points within the pathology of COVID-19 and Alzheimer’s disease may potentially provide new targets for therapeutic interventions, especially for the treatment of COVID-19 patients at risk of dementia. The bidirectional interplay between COVID-19 and Alzheimer’s disease was observed not just at the molecular level but also at the level of clinical symptomology and outcomes. Patients with Alzheimer’s disease are at a significantly higher risk of acquiring COVID-19, and once infected, they face a higher rate of hospitalization. Additionally, these patients are predisposed to not receiving proper treatment due to atypical presentation and cognitive decline, leading to a higher mortality rate compared to patients with either COVID-19 or dementia alone [9, 58, 60–62, 84]. Furthermore, a point of interest was that the elderly and males were more susceptible to acquiring either Alzheimer’s or COVID-19, whereas post-COVID-19 infection, the development of newly diagnosed Alzheimer’s dementia seems to prefer the elderly and females. The reasons are still not clear and therefore require further investigation [33].

The rapid spread of the SARS-CoV-2 virus led to the implementation of lockdown and restrictive measures all over the world. As a result, patients with Alzheimer’s disease reported worsening mood symptoms, such as loneliness, depression, hallucinations, anxiety, agitation, and aberrant motor behavior along with cognitive defects, such as memory loss and compromised recall ability [47, 64, 66–68, 70, 85, 86]. These studies underscore the need for further large-scale, robust studies to precisely understand the morbidity of patients with Alzheimer’s disease during lockdowns. Such research is crucial to identify the most prevalent and significant symptoms observed, enabling the introduction of highly tailored and specific mitigating measures.

Social connectivity/family bonding, as a means of overcoming loneliness, is one of the most important social measures that can delay the progression of dementia in patients with Alzheimer’s disease [87, 88] and thereby social connectedness and healthy relationships are protective factors for patients with Alzheimer’s disease [89–91]. The Harvard adult study, the longest longitudinal study on human life that followed 268 Harvard sophomores for a period of 80 years to understand what the key factors that lead to a longer and healthier life, reported that one of the most powerful indicators of good health and happiness is strong, trustworthy and satisfied relationships. The study also reported that one of the most important prognostic markers of death was loneliness and that most people who died early were lonely and socially unconnected [92]. This finding is supported by a recent study in the USA that showed adults living alone had a 32% increased risk of cancer death and this risk was higher (43%) in older individuals (age range 45–64) compared with middle-aged adults living with others [93]. Thus, strategies for mitigating loneliness appear beneficial in the outcomes of dementia and patients with Alzheimer’s disease. Hence, in a pandemic situation, patients and family members should be encouraged to regain social connectivity and (re)strengthen relationships with family members as a protective mechanism against their mental and physical decline. Moreover, further research is required to assess how training in improving social connectivity for Alzheimer’s disease patients can be optimally performed in a clinical setting during a pandemic possibly through the use of modern technology, such as AI and virtual 4D imaging [94]. Recent studies suggested that mindfulness meditation might also be a viable low-cost intervention to mitigate the psychological impact of COVID-19 and possibly future pandemics [95, 96]. Additionally, a multitude of studies within the literature have reported the beneficial effects of a good diet (presumably to maintain a healthy gut microflora), physical exercise, and group activities for patients with Alzheimer’s disease in maintaining their cognition and slowing the progression of the disease [97–99].

The adverse neuropsychological effects of lockdown and restrictive measures during the pandemic extended to the caregivers of Alzheimer’s disease patients as well. Caregivers reported increased anxiety and loneliness, as well as experiencing a greater care burden and stress. These factors impacted their ability to provide care and accurately assess the wellbeing of the Alzheimer’s disease patients they were caring for [11, 67, 73, 74]. However, caregivers reported that social support, close family relationships, interests, and hobbies were deemed beneficial in mitigating the emotional and psychological challenges brought on by the SARS-CoV-2 pandemic and lockdown [76]. Therefore, we propose the implementation of further education and training programs for caregivers. These programs would equip them with the necessary tools to ensure their own physical and mental wellbeing, as well as enable them to provide optimal care for their patients with Alzheimer’s dementia.

## Conclusion

There appears to be a significant overlap in the pathology and symptomology of Alzheimer’s disease and COVID-19. Alzheimer patients have an increased risk of acquiring COVID-19 infection and once infected, exhibit worsening clinical outcomes, including increasing morbidity and mortality. Similarly, elderly patients contracting SARS-COV-2 infection are more likely to develop neurological and psychiatric symptoms akin to dementia and Alzheimer’s disease. Furthermore, strict anti-social lockdown measures negatively impacted patients with Alzheimer’s disease and their caregivers including their ability to effectively provide care and accurately assess the wellbeing of their patients. Thus, additional safeguard measures in conjunction with pharmacological and non-pharmacological approaches are needed to protect the wellbeing of dementia patients and their caregivers in light of this pandemic.
